# Mackerel Trypsin Purified from Defatted Viscera by Supercritical Carbon Dioxide

**DOI:** 10.4061/2011/728082

**Published:** 2011-07-13

**Authors:** Byung-Soo Chun, Hideki Kishimura, Sitthipong Nalinanon, Sappasith Klomklao, Soottawat Benjakul

**Affiliations:** ^1^Department of Food Science and Technology, Pukyong National University, Busan 608-737, Republic of Korea; ^2^Research Faculty of Fisheries Sciences, Hokkaido University, Hakodate, Hokkaido 041-8611, Japan; ^3^Faculty of Agro-Industry, King Mongkut's Institute of Technology Ladkrabang, Choakhunthaharn Building, Choakhunthaharn Rd., Ladkrabang, Bangkok 10520, Thailand; ^4^Department of Food Technology, Faculty of Agro-Industry, Prince of Songkla University, Hat Yai, Songkhla 90112, Thailand; ^5^Department of Food Science and Technology, Faculty of Technology and Community Development, Thaksin University, Phattalung Campus, Phattalung 93110, Thailand

## Abstract

Viscera of mackerel (*Scomber* sp.) were defatted by supercritical carbon dioxide (SCO_2_) treatment. Trypsin (SC-T) was then extracted from the defatted powder and purified by a series of chromatographies including Sephacryl S-200 and Sephadex G-50. The purified SC-T was nearly homogeneous on SDS-PAGE, and its molecular weight was estimated as approximately 24,000 Da. *N*-terminal twenty amino acids sequence of SC-T was IVGGYECTAHSQPHQVSLNS. The specific trypsin inhibitors, soybean trypsin inhibitor and TLCK, strongly inhibited the activities of SC-T. The pH and temperature optimums of SC-T were at around pH 8.0 and 60°C, respectively, using *N* ^*α*^-*p*-tosyl-L-arginine methyl ester as a substrate. The SC-T was unstable below pH 5.0 and above 40°C, and it was stabilized by calcium ion. These enzymatic characteristics of SC-T were the same as those of other fish trypsins, especially spotted mackerel (*S. borealis*) trypsin, purified from viscera defatted by acetone. Therefore, we concluded that the SCO_2_ defatting process is useful as a substitute for organic solvent defatting process.

## 1. Introduction

Fish viscera are one of the sources of digestive enzymes that may have some unique properties of fascinate with both basic research and industrial applications. Their survival in waters required adaptation of their enzyme activity to low temperatures of their habitats. That is to say, fish proteinases have higher catalytic efficiency at low temperatures than those from warm-blooded animals [1, 2]. In addition, the strong positive correlation between the habitat temperature of marine fish and the thermostability of its trypsin has been demonstrated [3–11]. High activity at low temperatures and instability against heat, low pH, and autolysis of fish proteinases are interesting for some industrial applications [12]. Cod trypsin is already practically used in food production and cosmetics [13, 14]. Furthermore, Pacific cod and Atlantic cod trypsins were utilized as catalyst of enzymatic peptide synthesis [9, 15].

On the other hand, lipids in the tissue prevent from extracting, preparing, and purifying enzymes [16]. Conventional methods for the removal of lipids from materials involve cooking, pressing, and liquid extraction. On liquid extraction for enzyme preparation, it is usually used with organic solvents, such as hexane, ethanol, and acetone, and so forth [16, 17]. However, The removal of lipids with organic solvents causes protein denaturation and/or loss of functional properties [18]. Organic solvents are also highly flammable and are toxic for human health. Consideration of such factors has led investigators to apply supercritical fluid extraction techniques to the lipid separation [19]. Carbon dioxide (CO_2_) is a popular supercritical extractant particularly in food processing, flavor and aroma isolation, and pharmaceuticals manufacture, because CO_2_ is nontoxic and does not leave a residue. Supercritical CO_2_ (SCO_2_) has been used for extraction of oils from some marine organisms [20–22]. But, the aims of these studies were mainly the gain of oils rich in polyunsaturated fatty acids, especially EPA and DHA. So, the application of SCO_2_ for isolation of enzymes and production of quality protein meal from different sources should be examined.

Recently, we prepared a defatted powder of squid viscera treating with SCO_2_ and detected protease, lipase, and amylase activities in crude extract from the powder [18]. Next, we purified a phospholipase A_2_ from the starfish pyloric ceca defatted by SCO_2_ extraction process [23]. In this study, with the aim of utilization of fish trypsin for food industry, we purified a trypsin (SC-T) from the mackerel viscera powder treated by SCO_2_ defatting process and compared its enzymatic properties with those of other fish trypsins purified from the viscera defatted by acetone.

## 2. Materials and Methods

### 2.1. Materials

Mackerel (*Scomber* sp.) were caught off Busan, Republic of Korea. The mackerel viscera were collected from F & F Co., Busan, Republic of Korea, and the visceral waste was brought to the laboratory in iced condition. The CO_2_ (99.99% pure) was supplied by KOSEM, Korea. Sephacryl S-200 and Sephadex G-50 were purchased from GE Healthcare UK Ltd. (Amersham, UK). *N^*α*^*-*p*-Tosyl-L-arginine methyl ester hydrochloride (TAME) and Ethylenediaminetetraacetic acid (EDTA) were obtained from Wako Pure Chemicals (Osaka, Japan). 1-(L-trans-epoxysuccinyl-leucylamino)-4-guanidinobutane (E-64), soybean trypsin inhibitor, *N*-*p*-tosyl-L-lysine chloromethyl ketone (TLCK), and pepstatin A were purchased from Sigma Chemical Co. (St. Louis, Mo, USA).

### 2.2. Condition of Supercritical Fluid Defatting

The defatted powder of mackerel viscera was prepared as described by Chun et al. [23] using semibatch type of supercritical fluid extraction unit. The lipid extraction by SCO_2_ was performed at temperature of 45°C and pressure of 25 MPa. The total extraction time was 2.5 h. The SCO_2_ defatted powder was stored at −60°C until further analysis.

### 2.3. Purification of Mackerel Trypsin (SC-T) from SCO_2_ Defatted Powder

Trypsin was extracted by stirring from 10.0 g of defatted powder in 50 volumes of 10 mM Tris-HCl buffer (pH 8.0) containing 1 mM CaCl_2_ at 5°C for 3 h. The extract was centrifuged (H-200, Kokusan, Tokyo, Japan) at 10,000 xg for 10 min, and then the supernatant was concentrated by lyophilization and used as crude trypsin (50 mL). Ten milliliters of crude trypsin was applied for four times to a column of Sephacryl S-200 (3.9 × 64 cm) pre-equilibrated with 10 mM Tris-HCl buffer (pH 8.0) containing 1 mM CaCl_2_,and proteins were eluted (0.8 mL/min) with the same buffer. Each main trypsin fractions were gathered and concentrated by lyophilization. Then the concentrated fraction (10 mL) was applied to a Sephadex G-50 column (3.9 × 64 cm) pre-equilibrated with the above buffer, and proteins were eluted (0.7 mL/min) with the same buffer. A single trypsin fraction was pooled and used as purified trypsin (SC-T).

### 2.4. Assay for Trypsin Activity

Trypsin activity was measured by the method of Hummel [24] using TAME as a substrate. One unit of enzyme activity was defined as the amount of the enzyme hydrolyzing one micromole of TAME in a minute. The effect of inhibitors on trypsin was determined by incubating trypsin with an equal volume of proteinase inhibitor solution to obtain the final concentration designated (0.1 mM E-64, 1 mg/mL soybean trypsin inhibitor, 5 mM TLCK, 1 mM pepstatin A and 2 mM EDTA) [25]. After incubation of the mixture at 25°C for 15 min, the remaining activity was measured, and percent inhibition was then calculated. The pH dependencies of trypsin were determined in 50 mM buffer solutions [acetic acid-sodium acetate (pH 4.0–7.0), Tris-HCl (pH 7.0–9.0), and glycine-NaOH (pH 9.0–11.0)] at 30°C. The temperature dependencies of trypsin were determined at pH 8.0 and at various temperatures. The temperature and pH stabilities of trypsin were found by incubating enzyme at pH 8.0 for 15 min at a range of 20–80°C and by incubating the enzyme at 30°C for 30 min at a range of pH 4.0–11.0, respectively. The effect of CaCl_2_ on trypsin activity was found by incubating the enzyme at 30°C and at pH 8.0 in the presence of 10 mM EDTA or 10 mM CaCl_2_.

### 2.5. Sodium Dodecyl Sulfate-Polyacrylamide Gel Electrophoresis (SDS-PAGE)

SDS-PAGE was carried out using a 0.1% SDS-13.75% polyacrylamide slab gel by the method of Laemmli [26]. The gel was stained with 0.1% Coomassie Brilliant Blue R-250 in 50% methanol-7% acetic acid, and the background of the gel was destained with 7% acetic acid.

### 2.6. Analysis of Amino Acid Sequence

To analyze the *N*-terminal amino acid sequence of SC-T, the enzyme was electroblotted to a polyvinylidene difluoride (PVDF) membrane after SDS-PAGE. The amino acid sequence of the enzyme was analyzed by using a protein sequencer, Procise 492 (Perkin Elmer, Foster City, Calif, USA).

### 2.7. Protein Determination

The protein concentration was determined by the method of Lowry et al. [27] using bovine serum albumin as a standard.

## 3. Results and Discussion

### 3.1. Effect of SCO_2_ Defatting Process for Trypsin Activity

The viscera of mackerel (*Scomber *sp.) were treated by SCO_2_ to separate lipids on the condition of 40°C, 25 MPa, and 2.5 h. Since SCO_2_ extracted almost all oil from the squid viscera in the previous study, we adopted the condition to remove lipids from the mackerel viscera [18]. Trypsin was then extracted from 10.0 g of defatted powder by SCO_2_, and the crude enzyme was prepared. As shown in Table 1, the crude enzyme contained 1,390 mg of total protein and 1,049 U of total trypsin activity. Previously, we extracted trypsin from the pyloric ceca powder (13.9 g) of spotted mackerel defatted by acetone, and 3,633 mg of total protein and 3,270 U of total trypsin activity were detected in the crude enzyme solution [5]. Although these data were not compared directly, the yields of protein and trypsin activity per weight of acetone powder were approximately two times higher than those of SCO_2_ powder. However, the specific activity (0.8 U/mg) of crude enzyme in this study is almost the same as that (0.90 U/mg) in the previous study [5]. So, it is thought that the difference of total activity might come from the variation of specimen, and the defatting condition with SCO_2_ in this study would not cause significant denaturation of fish trypsin.

### 3.2. Purification of SC-T

**Figure 1:**
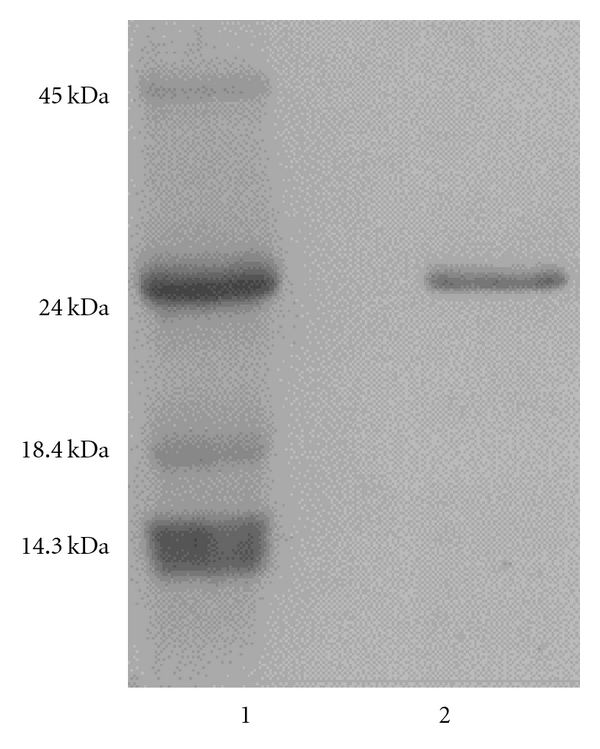
Electrophoresis of the trypsin (SC-T) purified from the mackerel (*Scomber *sp.) viscera defatted by SCO_2_. Electrophoresis was performed using a 0.1% SDS-13.75% polyacrylamide slab gel. (1) contains protein standards; ovalbumin (molecular weight, 45 kDa), bovine pancreatic trypsinogen (24 kDa), bovine milk b-lactoglobulin (18.4 kDa), and egg-white lysozyme (14.3 kDa). (2) contains mackerel trypsin (SC-T).

The SC-T was purified from the crude enzyme solution by two steps of chromatographies including Sephacryl S-200 and Sephadex G-50, which is the same purification procedure as that in the previous study [5]. The SC-T was consequently purified 48-fold with a high recovery (50%) from the crude enzyme solution (Table 1) and had a specific activity of 36 U/mg which is fairly higher than that of spotted mackerel trypsin [5]. In addition, the SC-T was found nearly homogeneous on SDS-PAGE (Figure 1), and its molecular weight was estimated as approximately 24,000, which is similar to that of spotted mackerel trypsin [5]. Furthermore, the *N*-terminal twenty amino acids sequence of SC-T was analyzed to be IVGGYECTPYSQPWTVSLNS that accords with that of spotted mackerel trypsin [5]. These results also show that the SCO_2_ defatting process in this study removes lipids in fish viscera as effectively as acetone defatting process for preparation of fish trypsin.

Crude enzyme extract usually contains various proteins, and sometimes fish trypsin is composed of some isozymes. In general, the purification of fish trypsin was carried out by the combination of some types of chromatography [28–30]. However, we achieved a high purification of a trypsin from the mackerel viscera powder defatted by acetone using only two steps of gel filtration [5]. So, in this study, we purified the SC-T by gel filtration according to the previous study.

### 3.3. Enzymatic Properties of SC-T

The SC-T was strongly inhibited by specific trypsin inhibitors (soybean trypsin inhibitor and TLCK), but E-64 (cysteine proteinase inhibitor), pepstatin A (aspartic proteinase inhibitor), and EDTA (metalloproteinase inhibitor) had no inhibitory effect on the activity of SC-T (Table 2).

**Figure 2:**
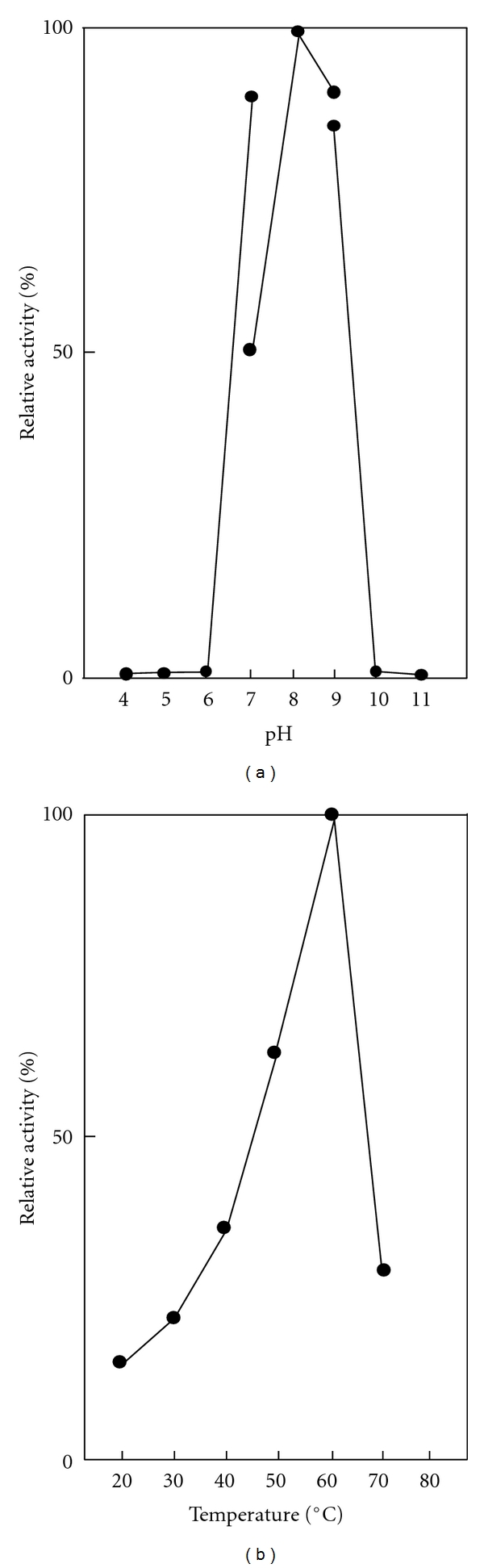
Effects of pH and temperature on the activity of SC-T. (a) The activity was determined in 50 mM buffer solutions [acetic acid-sodium acetate (pH 4.0–7.0), Tris-HCl (pH 7.0–9.0), and glycine-NaOH (pH 9.0–11.0)] at 37°C. (b) The activity was determined at pH 8.0 and at various temperatures.

The influence of pH on the SC-T activity is shown in Figure 2(a). The enzyme hydrolyzed TAME substrate effectively between pH 7.0 and 9.0, with an optimum around pH 8.0. The optimum pH of SC-T was the same as those of other fish trypsins [3–11, 31–38], but lower than those of bluefish (pH 9.5) [39] and Atlantic bonito (pH 9.0) [40]. Figure 2(b) shows the temperature dependencies of SC-T. The SC-T was active over a broad temperature range (20–70°C) with the optimum at about 60°C. Because mackerel is a temperate-zone fish, the SC-T possesses similar optimum temperature with other trypsins from temperate-zone fish, such as anchovy [3], true sardine [4], yellow tail [6], and jacopever [7]. The optimum temperature SC-T is slightly lower than those of tropical-zone fish (around 65°C) [33–35, 39, 40] but is evidently higher than those of frigid-zone fish trypsins (around 50°C) [4, 6–10].

**Figure 3:**
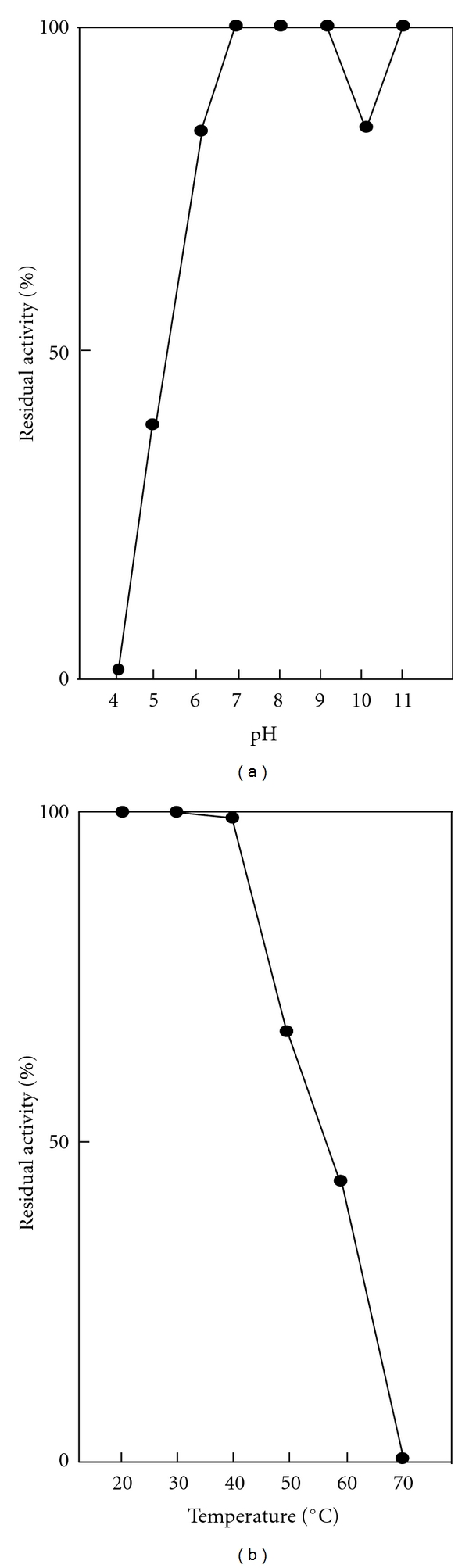
pH and temperature stability of SC-T. (a) The enzyme was kept at 30°C for 30 min and pH 4.0–11.0, and then the remaining activity at 30°C and pH 8.0 was determined. (b) The enzyme was kept at 20–70°C for 15 min and pH 8.0, and then the remaining activity at 30°C and pH 8.0 was determined.

The pH stability of SC-T is shown in Figure 3(a). The SC-T was stable at 30°C for 30 min in the pH range from pH 6.0 to 11.0. Unlike mammalian trypsins, diminished stability of the trypsin was more pronounced after exposure at acidic pH. Instability at acidic pH was also observed for other fish trypsins [1, 3–11, 32–35, 37, 39, 40]. For temperature stability, the SC-T was stable below 40°C, but the activity quickly fell over 50°C (Figure 3(b)). While the SC-T and other temperate-zone fish trypsins are relatively less stable than tropical-zone fish trypsins, they are obviously stable than frigid-zone fish trypsins [8, 10]. As described previously, there is a strong relationship between habitat temperature of marine fish and thermostability of their trypsins [8, 10].

**Figure 4:**
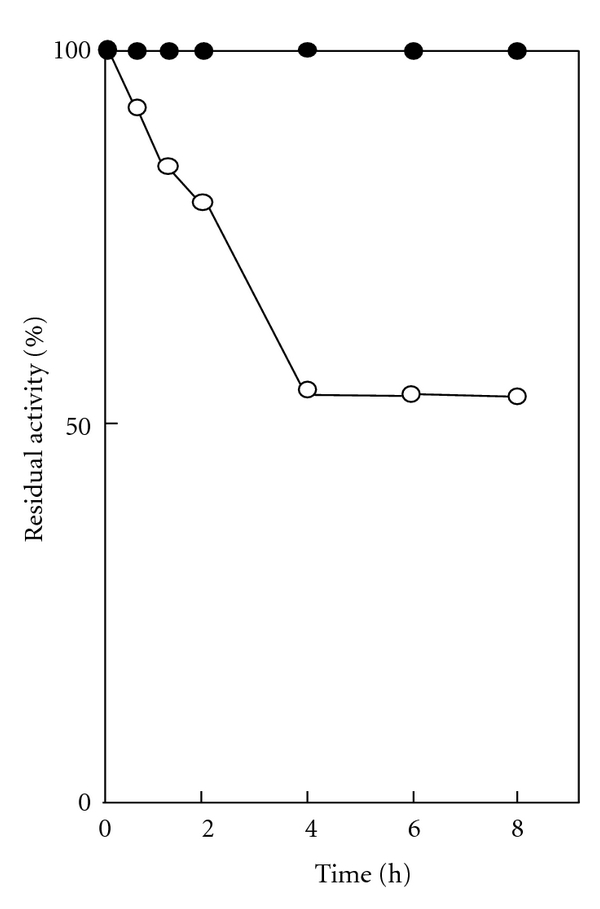
Effect of calcium ion on the stability of SC-T. The enzyme was kept at 30°C and pH 8.0 for 0–8 h in the presence of 10 mM CaCl_2_ (closed symbol) or 10 mM EDTA (open symbol), and then the remaining activity at 30°C and pH 8.0 was determined.

The effect of calcium ion on the stability of SC-T was then investigated. The stability of SC-T was enhanced by calcium ion (Figure 4). Similar results have been reported for various fish trypsins [1, 3–11, 33–35, 39, 40]. Bovine trypsinogen has two calcium-binding sites, and the primary site, with a higher affinity for calcium ions, is common in trypsinogen and trypsin and the secondary site is only in the zymogen [41, 42]. Occupancy of the primary calcium-binding site stabilizes bovine trypsin toward thermal denaturation or autolysis [41, 42]. In the previous paper, we described trypsin of arabesque greenling which also has the primary calcium-binding site [43]. The SC-T was stabilized by calcium ion from denaturation in this study. So, the result suggests that the SC-T may possess the primary calcium-binding site like bovine and arabesque greenling trypsins.

## 4. Conclusion

With the aim of utilization of fish trypsin for food industry, we purified a trypsin (SC-T) from mackerel (*Scomber *sp.) viscera powder treated by the SCO_2_ defatting process and compared its enzymatic properties with those of other fish trypsins purified from the viscera defatted by acetone. In this study, we adopted the condition of 40°C, 25 MPa, and 2.5 h to separate lipids from the viscera. Consequently, we could remove most of the lipids from the viscera and could extract considerable amount of trypsin from the defatted powder. The characteristics of purified SC-T were nearly the same as those of other fish trypsins, especially spotted mackerel trypsin. Therefore, we concluded that the SCO_2_ defatting process is useful as a substitute for the organic solvent defatting process.

## Figures and Tables

**Table 1:** Purification of trypsin (SC-1) from mackerel viscera defamed by SCO_2_.

Purification	Protein	Total	Specific	Purification	Yield
stages	(mg)	activity	activity	(fold)	(%)
		(U)	(U/mg)		
Crude enzyme	1,390	1,049	0.8	1	100
Sephacryl S-200	502	946	2	3	90
Sephadex G-50	15	547	36	48	50

**Table 2:** Effects of various inhibitors on the activity of SC-T^a^.

Inhibitors	Concentration	% Inhibition
Control	—	0
Soybean trypsin inhibitor	1 mg/mL	100
TLCK	5 mM	92
E-64	0.01 mM	0
Pepstatin A	0.01 mM	0
EDTA	2 mM	0

^a^ The enzyme solution was incubated with the same volume of inhibitor at 25°C for 15 min, and residual activity was analysed using TAME as a substrate for 5 min at pH 8.0 and 30°C.
